# Platelet-rich plasma inhibits Adriamycin-induced inflammation via blocking the NF-κB pathway in articular chondrocytes

**DOI:** 10.1186/s10020-021-00314-2

**Published:** 2021-06-25

**Authors:** Haijun Zhao, Weijie Zhu, Wude Mao, Chengkai Shen

**Affiliations:** Department of Joint Trauma Surgery, Qingdao Jiaozhou Central Hospital, No. 29 Xuzhou Road, Jiaozhou, 266300 Shandong People’s Republic of China

**Keywords:** PRP, Doxorubicin, Osteoarthritis, Chondrocyte, Inflammation, NF-κb

## Abstract

**Background:**

Previous studies showed that doxorubicin could lead to osteoarthritis (OA) by inducing chondrocyte inflammation and apoptosis. Besides, it is reported that platelet-rich plasma (PRP) could suppress the activation of inflammatory NF-κB signaling. Here, we aimed to determine whether PRP was able to exert a protective effect against doxorubicin-induced chondrocyte damages.

**Methods:**

To determine whether PRP protects chondrocytes against destabilization of the medial meniscus (DMM)-induced osteoarthritis, mice were treated with PRP and doxorubicin, and the cartilage destruction was observed through Safranin O-fast green staining and osteoarthritis scoring. ELISA assay was used to check the release of TNF-α and ILs. In vitro, we treated chondrocytes with doxorubicin and PRP; CCK-8 was used to measure cell viability. Western blot, real-time PCR, and ELISA were applied to check apoptosis-related signaling and inflammation-associated factors.

**Results:**

The results from the mouse model suggested that PRP attenuated doxorubicin-induced cartilage destruction in vivo. Doxorubicin promoted chondrocyte apoptosis while PRP ameliorated this damage. PRP inhibited doxorubicin-induced dysregulation of cell matrix-related factors, including SOX9, Col2A1, Col10A1, and Aggrecan, reduced protein levels of doxorubicin-induced inflammatory markers, COX-2, and iNOS, and blocked doxorubicin-induced phosphorylation of IκB and NF-κB in articular chondrocytes.

**Conclusions:**

PRP improved doxorubicin-induced damage on chondrocytes. This research might provide a new theoretical basis for the clinical treatment of osteoarthritis caused by doxorubicin.

**Supplementary Information:**

The online version contains supplementary material available at 10.1186/s10020-021-00314-2.

## Introduction

Osteoarthritis (OA) is a prevalent joint disorder globally, occurring in about 20% of people aged over 60-year old, and severely affecting the daily life of those patients (Chen et al. 2017). During the process of OA, cartilage destruction usually occurs in joints of knees, hands, and spine, slowly damaging articular cartilage and disabling the function of whole joints (Sherwood 2019). The crucial pathological factors of OA involve aging, obesity, joint instability, and especially joint inflammation (Sherwood 2019). It is suggested that inflammation functions at the early stage of OA drive various pathological changes during its progression, including articular cartilage degeneration (Robinson et al. 2016). Articular cartilage only contains one type of cells, namely chondrocytes, filled with the extracellular matrix that mainly constructed by collagens (Liao et al. 2017; Guilak et al. 2018). It was indicated that chondrocytes undergo apoptotic cell death during OA pathogenesis, and the collagens could be degraded by matrix metalloproteinases (MMPs), such as MMP3 and MMP13, which were upregulated in OA samples as reported in previous studies (Lepetsos et al. 2019). These studies indicated that targeting cell apoptosis and matrix remodeling might be effective strategies for OA therapy.

Despite the high prevalence of OA and its severe impacts on human health, no treatment has been approved by FDA to prevent or ameliorate OA progression, and surgical joint replacement is the only effective strategy for end-stage OA (Bannuru et al. 2015; Gregori et al. 2018). Hence, it is urgent to develop novel and effective therapeutic manners for OA. Platelet-rich plasma (PRP) is autologous plasma with concentrated platelets, which would secrete various growth factors, cytokines, chemokines, and other functional molecules when activated (Xie et al. 2014). In recent years, an increasing number of studies have focused on PRP application to ameliorate the inflammatory state during OA (Xu et al. 2017; Fukawa et al. 2017; Fotouhi et al. 2018; Chouhan et al. 2019). Fotouhi et al. (2018) reported that intra-articular PRP injections showed multiple favorable results for patients with OA. Another study on the guinea pig model of knee OA suggested that multiple PRP injections provided better therapeutic outcomes than a single injection (Chouhan et al. 2019). Here, we tried to further elucidate the function of PRP for OA treatment.

Doxorubicin is an effective antibiotic drug with a broad anti-tumor spectrum, although its clinical application is limited by multiple side effects, including generating reactive oxygen species and nitric oxide and causing cell apoptosis (Kalivendi et al. 2001; Mukhopadhyay et al. 2009; Speth et al. 1988; Rivankar 2014). Kumagai et al. (2012) also proposed that doxorubicin could induce apoptosis of chondrocytes by causing cell volume decrease. Yet, the profound mechanisms underlying doxorubicin-induced apoptosis of chondrocytes and whether PRP administration could ameliorate this doxorubicin-caused damage are still unclear.

In this study, we performed in vitro and in vivo experiments to evaluate whether PRP was able to reverse doxorubicin-induced damage on chondrocytes, with the hope to provide novel insights into developing OA therapies.

## Materials and methods

### Animal model

All animal experiments in this study were conducted strictly following the legislation of the use and care of laboratory animals in China and approved by Qingdao Jiaozhou Central Hospital (No. 632278#ERF). 12-week-old male C57BL/6 mice were housed in a designated mouse facility operating at a controlled temperature (24 °C) and a circadian rhythm of 12 h and had free access to clean water and foods. These mice were randomly divided into the indicated groups in corresponding experiments. OA was induced in mice by destabilizing the medial meniscus (DMM) of the knees. One week after surgery, the mice received doxorubicin (10 mg/kg) injection and/or intra-articular injection of 15 μL PRP every week. After 4 weeks, the mice were euthanized, and the knee joints and plasma were collected (Hu et al. 2020).

### PRP extraction

PRP was isolated from the blood of euthanized mice. In brief, whole blood was collected using heparin-containing sterile syringes and centrifuged for 10 min at 1800 rpm. The bottom layer with red blood cells was discarded, and the upper and intermediate layers were collected and centrifuged again at 3000 rpm for 6 min. After carefully removing the upper 75% supernatant, the remainder was resuspended in DMEM/F12 media and filtered (Bos-Mikich et al. 2018) to obtain PRP. PRP concentration was determined according to TGF-β1 concentration via ELISA (Additional file 1: Fig. S1).

### Enzyme-linked immunosorbent assay (ELISA)

The serum levels of inflammatory factors TGF-β1, TNF-α, IL-1β, and IL-6 were checked using ELISA following the manufacturer’s instructions. IL-6 (M6000B), TGF-β1 (DB100B), TNF-α (MTA00B), IL-1β (MLB00C) were purchased from R&D systems (Minneapolis, MN).

### Histopathologic analysis

The knee joint samples were collected and fixed in 4% paraformaldehyde for 24 h. The bones were placed in 10% EDTA for 1 month to perform decalcification, embedded with paraffin, prepared as 5 μm thick sections, and stained with Safranin O-fast green staining kit (G1371, Solarbio, China) following manufacturer’s instruction. The images were captured and calculated. The scores of the femur and tibia were summed and presented as the OARSI score for each sample.

### Cell culture

Mice were sacrificed, and the total articular cartilage was isolated from the femur head, cut into pieces, and digested with 1.5 mg/mL pronase for 2 h and collagenase II under shaking at 37 °C for 8 h. Cells were then filtered through a 70 μm cell strainer and washed 3 times with PBS. Subsequently, the obtained chondrocytes were placed in culture plates and maintained in DMEM/F12 media (Gibco, Thermo, MA, USA) containing 10% fetal bovine serum (FBS, Gibco), 100 U/mL penicillin, and 100 μg/mL streptomycin. The media were changed every 2 days. After preparation of chondrocytes from joint articulation, toluidine blue staining and collagen type II immunohistochemical staining were carried out to identify the purity of these chondrocytes (Additional file 1: Fig. S1).

Bone marrow-derived macrophages (BMMs) were extracted from the bone marrow of the femur and tibia of mice and cultured in Dulbecco’s modified Eagle medium (DMEM) supplemented with 10% FBS with M-CSF (10 ng/mL). BMMs were differentiated in a humidified chamber at 37 °C with 5% CO_2_ with changing cell culture media every 3 days until they became confluent. Afterward, cells were plated for experimental treatments.

ATDC5 cells were cultured in Alpha modification of Eagle’s media containing 10% FBS, 100 U/mL penicillin, and Insulin-Transferrin-Selenium solution. All cells were cultured at 37 °C in a humidified incubator with 5% CO_2_.

### Cell viability assay

The proliferation ability of cardiomyocytes was analyzed by CCK-8 kit. In brief, cells were digested and plated into 96-well plates with a density of 1 × 10^3^ cells per well overnight and treated with doxorubicin or PRP at the indicated concentration for 24 h. At the treatment, 10 μL of CCK-8 (Roche, Basel, Switzerland) was added to each well, and cells were continuously incubated for 1 more hour at the incubator. Then the absorbance values at 450 nm was detected by an absorbance reader.

### Western blot

Cells were lysed in 1× ice-cold RIPA lysis buffer (Beyotime, Shanghai, China) and centrifuged to discard the deposition. The protein concentration was determined using BCA kits (TakaRA, Dalian, China). A total of 35 μg proteins were separated on 10% SDS-polyacrylamide gel (PAGE) and transferred to polyvinylidene difluoride (PVDF) membranes. The membranes were blocked for 1 h at room temperature with 5% nonfat milk dissolved in 1× TBST and incubated with specific primary antibodies at 4 °C overnight. Then the membranes were further incubated for 2 h at room temperature with the corresponding secondary antibodies. After that, labeled proteins were visualized by an ECL kit (Invitrogen, CA, USA) and captured using Gel imaging system (BD, USA). Primary antibodies against p-NF-κB (3033), NF-κB (3032), p-IκBα, (8219), IκBα (9242) were purchased from Cell Signaling Technology (MA, USA) and against β-actin (sc-1616), COX-2 (sc-376861), Sox9 (SC-166505), iNOS (SC-7271), PARP (SC-74470) and cleaved PARP (SC-56196) were purchased from SantaCruz Biotechnology (CA, USA) and diluted following the manufacturers’ protocols.

### RNA extraction and real-time PCR

Total RNAs were extracted using TRIzol Reagent (Invitrogen, CA, USA) following the manufacturer’s protocols after indicated treatments. Their concentration was determined spectrophotometrically at 260 nm (Thermo-Scientific NanoDrop 2000), and their quality and purity were verified by A260/A280 ratio (1.8–2.2). Then RNAs were reverse transcribed to cDNA using a cDNA Synthesis Kit (Takara Biotechnology, Japan). Quantitative real-time PCR (qRT-PCR) was performed using the SYBR Green SuperMix kit (BD, USA) to detect gene expression following the manufacturer’s protocols. Small endogenous nucleolar U6 snRNA and GAPDH were used as the internal controls for normalizing miR-125a-5p and MTFP1, respectively. Gene expression level was determined using the 2^−ΔΔCt^ method. The primers used in this study were MMP1 forward 5ʹ-ATACGTTGGTGGTGTTGTAATGT-3ʹ and reverse 5ʹ-GTCA CCTCTTTGGATGCCATAAA-3ʹ, MMP3 forward, 5ʹ-TTAAAGACAGGCACTTTTG GCG-3ʹ and reverse 5ʹ-CCCTCGTATAGCCCAGAACT-3ʹ, MMP13 forward 5ʹ-CTT TGGCTTAGAGGTGACTGG-3ʹ and reverse 5ʹ-AGGCACTCCACATCTTGGTTT-3ʹ, TNF-α forward 5ʹ-TGGAACACGTCGTGGGATAATG-3ʹ and reverse 5ʹ-GGCAGAC TTTGGATGCTTCTT-3ʹ, IL-6 forward 5ʹ-GGCGGATCGGATGTTGTGAT-3ʹ and reverse 5ʹ-GGACCCCAGACAATCGGTTG-3ʹ, Col2A1 forward 5ʹ-CCTCAAGGCA AAGTTGGTCCT-3ʹ and reverse 5ʹ-CTCCCGTCTCACCGTCTTTT-3ʹ, Col10A1 forward 5ʹ-GGTGTGAATGGGCGGAAAG-3ʹ and reverse 5ʹ-GCTTCCCAATACCTT CTCGTC-3ʹ, as well as Aggrecan forward 5ʹ-CCCAGGATAAAACCAGGCAG-3ʹ and reverse 5ʹ-CGGCCAAGGGTTGTAAATGG-3ʹ.

### Statistical analysis

The data were presented as the mean ± SD. Student t test and One-way ANOVA method followed by Bonferroni post hoc test were used to assess the difference between two groups and multiple groups, separately. A p < 0.05 was considered statistically significant.

## Results

### PRP reduced doxorubicin exposure-increased susceptibility to osteoarthritis

**Fig. 1:**
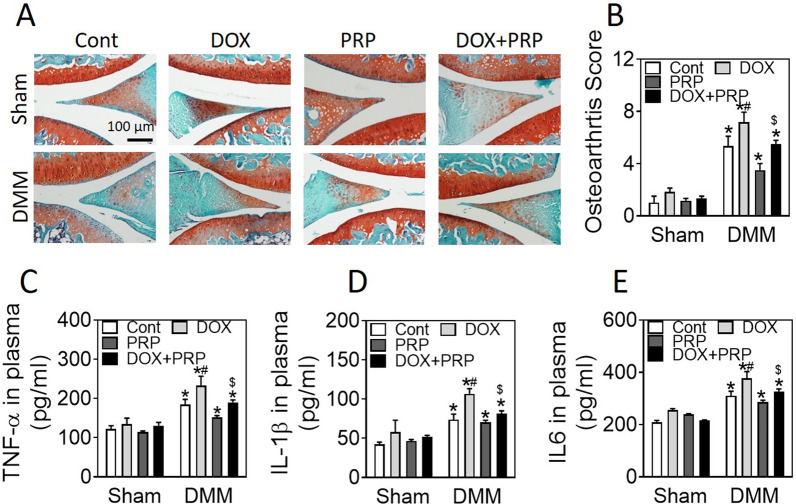
PRP reduces doxorubicin exposure-increased susceptibility to osteoarthritis. The 12-week-old C57BL/6J mice were under DMM surgery for 1 week and received doxorubicin (10 mg/kg) injection and/or intra-articular injection of 15 μL PRP every week. After 4 weeks, mouse knee joints were collected. **A**, **B** Representative images of Safranin O/fast green staining (**A**) and quantification (**B**). **C**–**E** Plasma levels of TNF-α (**C**), IL-1β (**D**), and IL6 (**E**). n = 6. *p < 0.05, vs sham of the same treatment; ^#^*p* < 0.05, vs control of the same DMM treatment; ^$^*p* < 0.05, vs doxorubicin of the same DMM treatment

To investigate whether PRP is able to exert a protective effect against doxorubicin-induced inflammation of chondrocytes, we treated mice with PRP and doxorubicin. As shown in Fig. 1A, B, doxorubicin treatment significantly degraded articular cartilage, and PRP injection protected mice against doxorubicin-induced osteoarthritis. In line with the pathological results, ELISA also revealed that doxorubicin exposure increased TNF-α, IL-1β, and IL6 protein levels, whereas PRP administration suppressed these increases (Fig. 1C–E). Together, these data suggested that PRP injection ameliorated doxorubicin exposure-induced osteoarthritis, probably through suppressing inflammation.

### PRP inhibited doxorubicin-induced chondrocyte apoptosis

**Fig. 2:**
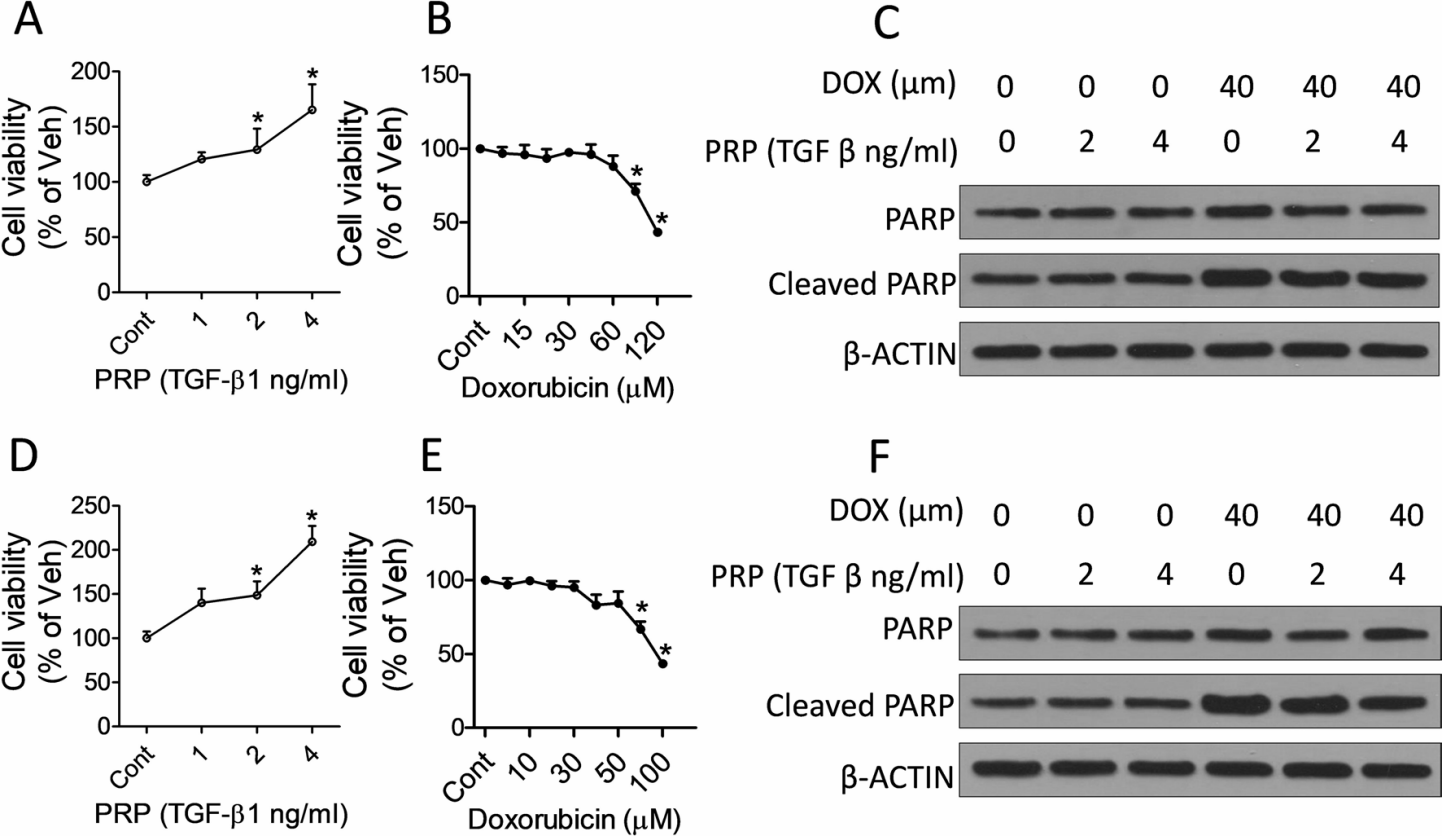
PRP inhibits doxorubicin-induced chondrocyte apoptosis. ATDC5 cells and primary articular chondrocytes were treated with elevated concentrations of PRP and 40 doxorubicin for 24 h. **A**, **B** CCK-8 assay to determine ATDC5 viability after PRP (**A**) or doxorubicin (**B**) treatment. **C** Western blot assay to detect protein levels of PARP and cleaved PARP in ATDC5 cells. **D**, **E** CCK-8 assay to determine the viability of primary articular chondrocyte cells treated with PRP (**D**) or doxorubicin (**E**). **F** Western blot assay to detect protein levels of PARP and cleaved PARP in primary articular chondrocyte cells. At least three independent experiments were performed. *p < 0.05 vs control

As the extract from total blood, PRP is considered a cocktail of growth factors, among which TGF-β1 is the most abundant, presentative ingredient. In this study, we determined PRP concentration by evaluating TGF-β1 level and investigated the proliferation of ATDC5 cells and primary articular chondrocytes. The results showed that 2 ng/mL PRP treatment enhanced proliferation of ATDC5 cells (Fig. 2A) and primary articular chondrocytes (Fig. 2D) compared with the control and lower concentration PRP treatment. We next evaluated the potential of doxorubicin to affect cell viability and apoptotic cell death. As shown in Fig. 2B, E, treatment with lower than 40 μM doxorubicin for 24 h had no significant effect on the viability of ATDC5 cells (Fig. 2B) and primary articular chondrocytes (Fig. 2E) compared with the control group. Hence, the maximum doxorubicin concentration used for subsequent experiments was 40 μM. We treated cells with doxorubicin and different concentrations of PRP for 24 h, and found that doxorubicin treatment increased the apoptosis-related marker PARP and cleaved-PARP, and PRP treatment inhibited doxorubicin-induced PARP cleavage in ATDC5 (Fig. 2C) and primary articular chondrocytes (Fig. 2F). These results suggested that PRP inhibited doxorubicin-induced chondrocyte apoptosis.

### PRP suppressed doxorubicin-induced inflammation

**Fig. 3:**
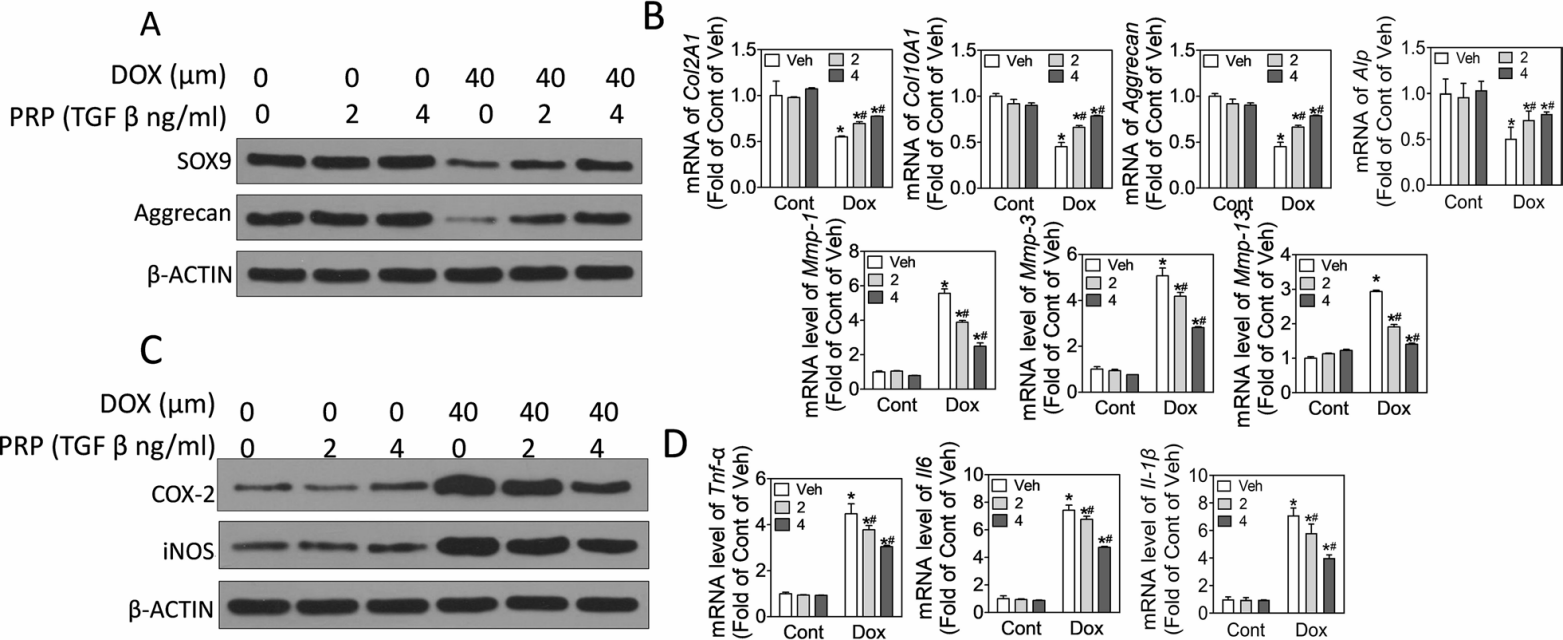
PRP suppresses doxorubicin exposure-induced inflammation. ATDC5 cells were treated with PRP and doxorubicin for 24 h. **A** Western blot results showing the protein levels of SOX9, Aggrecan, and β-actin. **B** Real-time PCR to detect mRNA levels of Col2A1, Col10A1, Aggrecan, ALP, MMP-1, MMP-3, and MMP-13. **C** Western blot to exam the protein levels of inflammatory factors COX-2 and iNOS, and β-actin. **D** Real-time PCR to detect mRNA levels of TNF-α, IL-6, and IL-1β. At least three independent experiments were performed. *p < 0.05 vs control of the same doxorubicin treatment; ^#^*p* < 0.05 vs vehicle of the same TGFβ treatment

To investigate the role of PRP on inflammation, we examined cell matrix degradation and levels of several critical inflammatory factors. As shown in Fig. 3A, doxorubicin exposure for 24 h notably suppressed the levels of SOX9, Col2A1, Col10A1, and Aggrecan, whereas this suppression was attenuated by PRP at a dose-dependent manner (Fig. 3A, B, p < 0.05). As expected, PRP treatment reversed elevated levels of MMP-1, MMP-3, and MMP-13, the potent enzymes for cartilage matrix degradation compared with doxorubicin treatment alone (Fig. 3B, p < 0.05). Furthermore, doxorubicin exposure increased the levels of inflammatory factors COX-2 and iNOS (Fig. 3C, p < 0.05) and other inflammatory indicators, such as TNF-α and IL-6 (Fig. 3D, p < 0.05). Moreover, PRP inhibited the effects of doxorubicin on these inflammatory biomarkers (Fig. 3D, p < 0.05). These data indicate that PRP could improve chondrocyte damage via suppressing inflammatory biomarkers elevated by doxorubicin exposure.

### PRP inhibited doxorubicin-induced inflammation via blocking NF-κB pathway

**Fig. 4:**
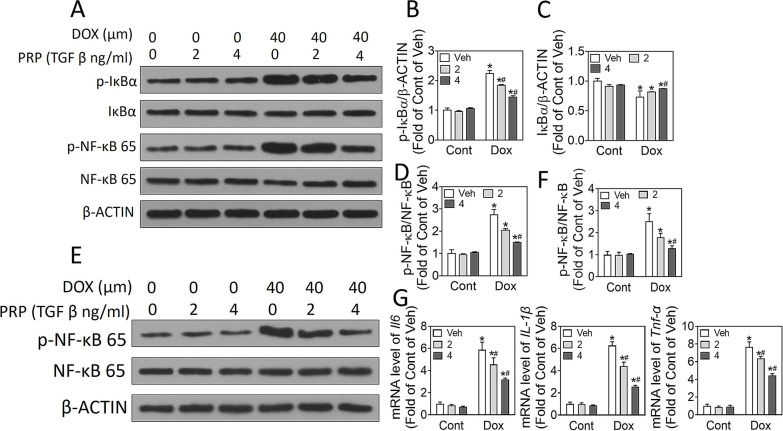
PRP inhibits doxorubicin-induced inflammation via blocking NF-κB pathway. ATDC5 cells were treated with PRP and doxorubicin for 24 h. **A** Western blot to exam the protein levels of p-IκBα, IκBα, p-NF-κB 65, NF-κB 65, and β-ACTIN. **B**–**D** Quantitation of the immunoblots. At least three independent experiments were performed. **E** BMM cells were treated with PRP and doxorubicin for 24 h. Western blot results of the protein levels of p-NF-κB, NF-κB, and β-actin. **F** Quantitation of the immunoblots. **G** Real-time PCR to detect mRNA levels of IL-6, IL-1β, and TNF-α. *p < 0.05 vs control of the same doxorubicin treatment; ^#^p < 0.05 vs vehicle of the same TGF β treatment

**Fig. 5:**
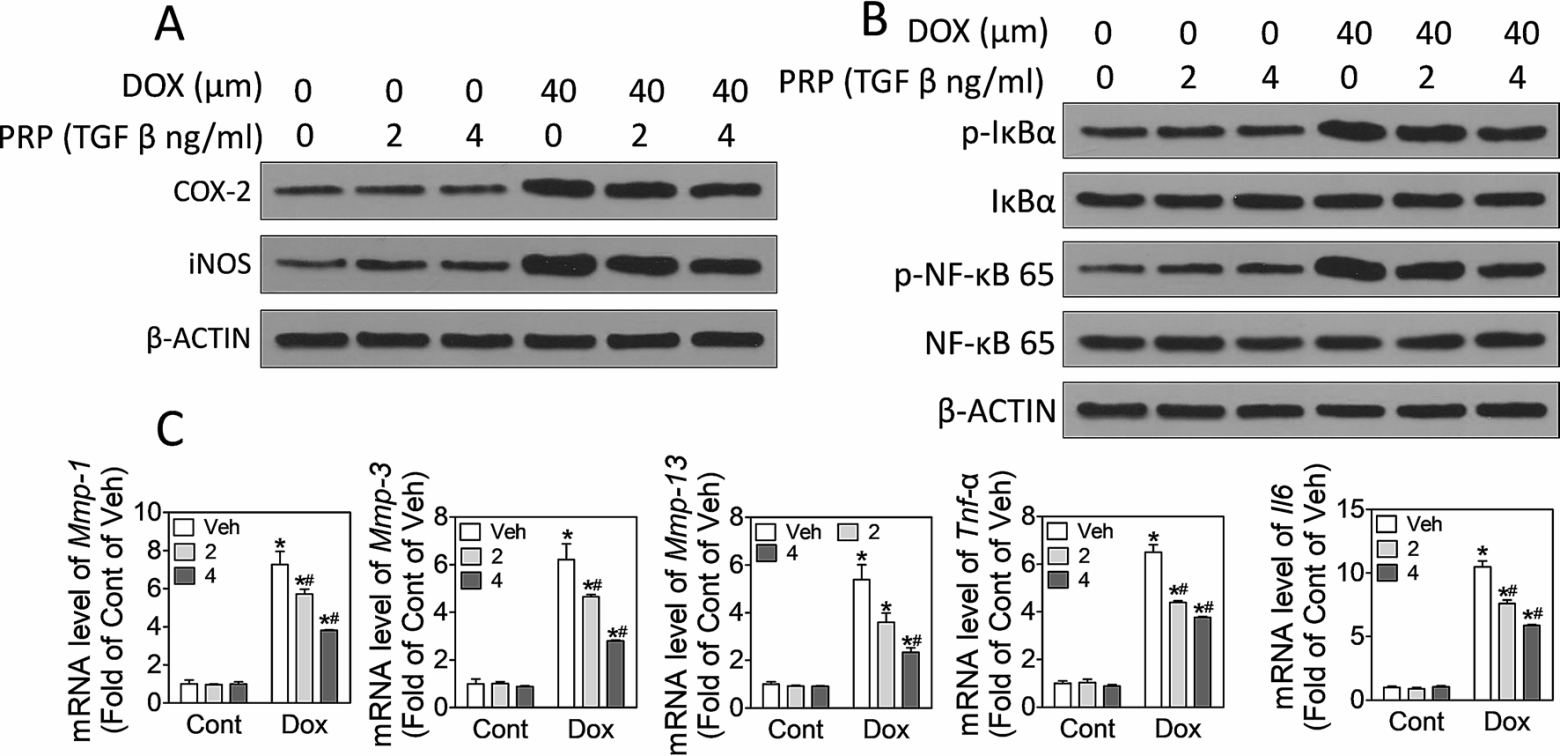
PRP inhibits doxorubicin-induced inflammation in SW1353. SW1353 were treated with PRP and doxorubicin for 24 h. **A**, **B** Western blot to detect protein levels of COX-2, iNOS, p-IκBα, IκBα, p-NF-κB 65, NF-κB 65, and β-actin. **C** mRNA levels of MMP-1, MMP-3, MMP-13, TNF-α, and IL-6. At least three independent experiments were performed. *p < 0.05 vs control of the same doxorubicin treatment; #*p* < 0.05 vs vehicle of the same TGFβ treatment

To further elucidate mechanisms underlying PRP-suppressed inflammation under doxorubicin administration, we detected the NF-κB signaling pathway. The results showed that exposure to 40 μM doxorubicin activated NF-κB pathway, with notably elevated IκBα phosphorylation and NF-κB translocation to the nucleus, and these changes were suppressed by PRP in a dose-dependent manner (Fig. 4A–D, p < 0.05) in ATDC5. Moreover, PRP also reversed doxorubicin-induced NF-κB activation and elevated IL-6, IL-1β and TNF-α levels in BMMs (Fig. 4E–G). Hence, we hypothesized PRP protected chondrocytes from doxorubicin-induced inflammation by inhibiting NF-κB signaling pathway. We further explored whether the NF-κB-dependent regulatory axis is also involved in the protective effects of PRP on primary articular chondrocyte cells. Consistent with the results from ATDC5, PRP remarkably reversed doxorubicin-elevation of inflammatory factors through suppressing NF-κB signaling activation (Fig. 5A, B, p < 0.05). Besides, PRP treatment dose-dependently ameliorated (Fig. 5C, p < 0.05) doxorubicin-induced cartilage matrix degradation, as manifested by reducing doxorubicin-induced elevation of MMP-1, MMP-3, MMP-13, and inflammatory factors TNF-α and IL-6 in primary articular chondrocytes cells (Fig. 5C, p < 0.05). In addition, because PRP contains many growth factors with the most abundant TGF-β1, we wondered whether TGF-β1 alone is enough to explain the effects of PRP on chondrocytes. Therefore, we evaluated the effects of TGF-β1 on the PARP cleavage and NF-κB activation. The results showed that TGF-β1 blocked PARP cleavage and NF-κB activation in ATDC5, SW1353, and BMMs (Additional file 2: Fig. S2), indicating that PRP inhibits doxorubicin-induced inflammation via blocking NF-κB pathways.

## Discussion

As a potent anti-cancer medicine, doxorubicin was widely used in clinical studies. However, its side effects severely restricted its application (Speth et al. 1988; Rivankar 2014). Numerous studies have indicated doxorubicin could cause normal cell death in various cell types, including chondrocytes (Kumagai et al. 2012). Our study also showed that doxorubicin treatment notably decreased the thickness of articular cartilage in the DMM model and elevated chondrocyte apoptosis and cleaved PARP protein level in vitro. These data confirmed the apoptosis-promoting role of doxorubicin during OA progression.

Early studies on OA stated that cartilage degradation could be independent of inflammatory response. However, numerous studies have proposed that low-grade and chronic inflammation at early stage remarkably promotes OA progression (Robinson et al. 2016; Chang et al. 2019; Shen et al. 2017). Hence, more therapeutic approaches are being developed to directly target the components of inflammatory signaling, among which the intra-articular PRP injection seems to be accompanied with much favorable outcomes (Fotouhi et al. 2018; Smith 2016; Xie et al. 2015).

The therapeutic effects of PRP on OA are considered mainly mediated by the anabolic growth factors and anti-inflammatory mediators through activating collagen synthesis in chondrocytes and simultaneously preventing chondrocyte apoptosis (Kennedy et al. 2018). Our study suggested that PRP injection notably attenuated doxorubicin-caused cartilage degeneration in the DMM model and suppressed levels of inflammatory factors TNF-α, IL-1β, and IL-6. We found that 40 μM doxorubicin treatment did not affect cell viability. Thus, it was used in subsequent in vitro studies. In vitro experiments further revealed PRP administration suppressed Cox2 and iNOS expression in doxorubicin-treated chondrocytes, consistent with our previous results that doxorubicin-induced cell apoptosis is associated with reactive oxygen species (ROS) production, MMP gene expression, and superoxide generation. Administration of PARP inhibitors and oxidase inhibitors could attenuate doxorubicin-induced cell death (Kalivendi et al. 2001; Mukhopadhyay et al. 2009; Mizutani et al. 2005). It has been reported that protein degradation induced by ubiquitin ligases is important in this system (Brigant et al. 2020). We, therefore, speculated that ubiquitin ligases might be involved in the underlying mechanism for apoptosis in chondrocytes induced by doxorubicin.

Sox9, Aggrecan, and type II collagen are produced by normal chondrocytes to facilitate chondrogenesis in joints and are regarded as particular chondrogenic biomarkers (Carballo et al. 2017). SOX9 functions as a crucial transcriptional factor at the early stage of chondrogenesis and is capable of activating the expression of specific cellular matrix proteins, including type II collagen (Col2A) and Aggrecan (Sanchez et al. 2017; Henrotin et al. 2007; Rousseau and Garnero 2012). These proteins exert important functions to maintain the biological structure of articular cartilage (Gu et al. 2014). Here, we found decreased expression of SOX9 and Aggrecan and impaired transcription of Col2A1, Col10A1 and Aggrecan genes under doxorubicin treatment. However, PRP could notably ameliorate the damaged matrix proteins, which further supported the favorable function of PRP in OA therapy.

NF-κB is a classic transcriptional factor during numerous inflammatory responses in multiple diseases, including OA (Saito and Tanaka 2017). Previous studies have revealed that NF-κB signaling upregulates the expression of MMPs, the enzymes that directly degrade collagens, leading to chondrocyte hypertrophy and formation of inflammatory OA (Lepetsos et al. 2019). NF-κB signaling is also critical for the expression of various inflammatory factors, including iNOS, TNF-α, and ILs (Liu et al. 2017). Our in vitro and in vivo studies showed that PRP administration notably reversed doxorubicin-induced elevation of inflammatory factors and collagen degradation during OA. We wonder if these anti-inflammatory functions of PRP are associated with NF-κB signaling. As expected, in both primary chondrocytes and ATDC5 cells, PRP treatment significantly suppressed doxorubicin-activated IκB phosphorylation and NF-κB, which could inhibit NF-κB translocation from cytoplasm to nucleus and expression of various inflammatory factors.

## Conclusion

**Fig. 6:**
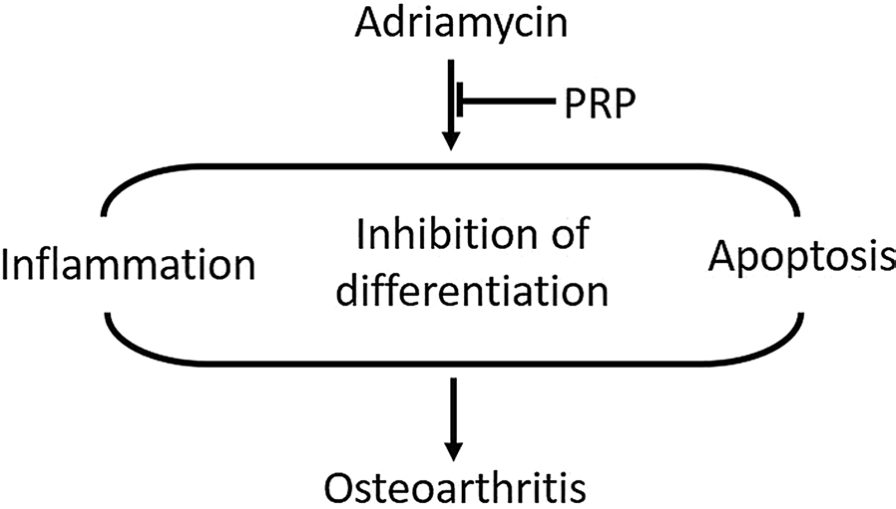
Proposed mechanism of PRP inhibiting doxorubicin-induced osteoarthritis. PRP prevented arthritis by inhibiting doxorubicin-induced chondrocyte differentiation, apoptosis, and inflammation

In conclusion, our study showed that the platelet-rich plasma (PRP) collected from blood exhibited favorable effects against doxorubicin-caused damages to articular cartilage in vivo. PRP suppressed doxorubicin-induced chondrocyte apoptosis and matrix degradation by attenuating NF-κB signaling and expression of downstream inflammatory factors iNOS, TNF-α, and ILs (Fig. 6). Our findings may provide more supportive evidence for the application of PRP to OA therapy.

## Supplementary Information

**Additional file 1: Figure S1.** Toluidine blue staining and collagen type II immunohistochemical staining of the mouse chondrocytes.

**Additional file 2: Figure S2.** TGF-β1 is the crucial factor in PRP responsible for its preventive effects on arthritis. Shown are the Western blot results of PARP, cleaved PARP, β-actin, p-NF-κB, and NF-κB in ATDC5 (A), primary articular chondrocytes cells (C), SW1353 (E) and BMMs (H) treated with PRP, doxorubicin, and TGF-β1 for 24 h, % of PRP action is the effect of TGF-β1 and other in ATDC5 (B), primary articular chondrocytes cells (D), SW1353 (F) and BMMs (H).

## Data Availability

The data that support the findings of this study are available on request from the corresponding author Haijun Zhao, Department of Joint Trauma Surgery, Qingdao Jiaozhou Central Hospital, No. 29 Xuzhou Road, Jiaozhou City, Shandong Province, 266300, P. R. China. Email: HaijunZhaoJiaozhou@163.com. The data are not publicly available due to their containing information that could compromise the privacy of research participants.
