# Supplementary figures and images for: Platelet-rich plasma inhibits Adriamycin-induced inflammation via blocking the NF-κB pathway in articular chondrocytes
Source: Mol Med. 2021 Jun 25;27:66. doi: 10.1186/s10020-021-00314-2 (PMC8229346; doi:10.1186/s10020-021-00314-2)

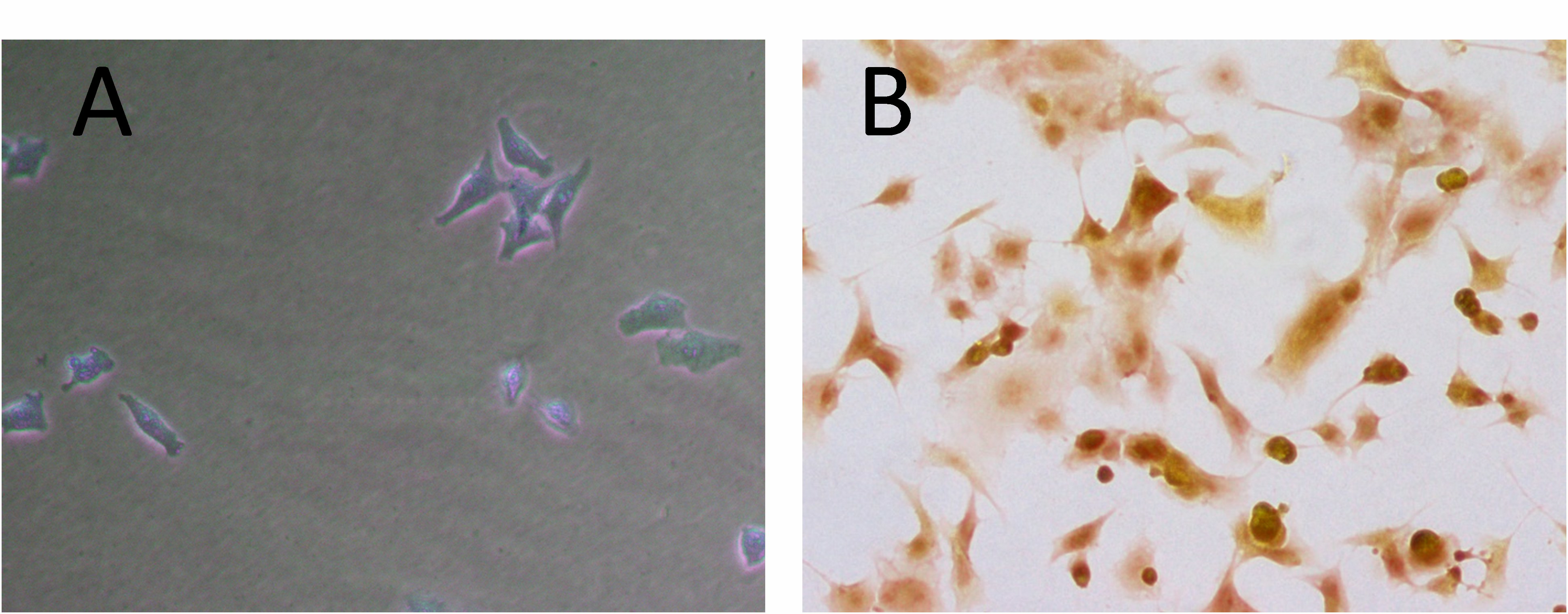

Supplement: Supplementary file 1 — Additional file 1: Figure S1. Toluidine blue staining and collagen type II immunohistochemical staining of the mouse chondrocytes. [file 10020_2021_314_MOESM1_ESM.tif]

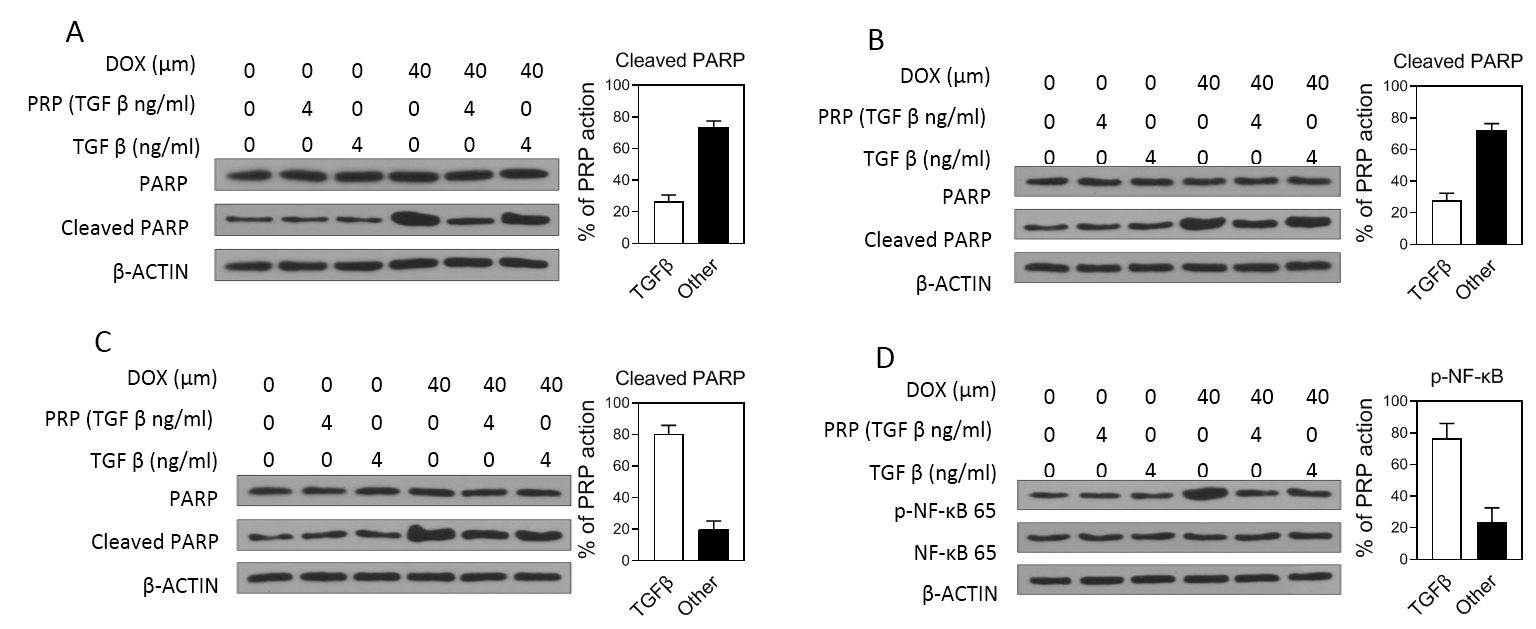

Supplement: Supplementary file 2 — Additional file 2: Figure S2. TGF-β1 is the crucial factor in PRP responsible for its preventive effects on arthritis. Shown are the Western blot results of PARP, cleaved PARP, β-actin, p-NF-κB, and NF-κB in ATDC5 (A), primary articular chondrocytes cells (C), SW1353 (E) and BMMs (H) treated with PRP, doxorubicin, and TGF-β1 for 24 h, % of PRP action is the effect of TGF-β1 and other in ATDC5 (B), primary articular chondrocytes cells (D), SW1353 (F) and BMMs (H). [file 10020_2021_314_MOESM2_ESM.tif]
